# Coding-complete genome sequence of a largemouth bass virus isolate from *Micropterus salmoides* collected in China

**DOI:** 10.1128/mra.00101-26

**Published:** 2026-04-20

**Authors:** Hengkang He, Hao Huang, Yang Zhang, Xiaogang Yang, Meisheng Yi, Kuntong Jia

**Affiliations:** 1 School of Marine Sciences, Sun Yat-Sen University 26469 , Guangzhou, China; 2 Guangdong Provincial Key Laboratory of Marine Resources and Coastal Engineering, Guangzhou, China; Queens College Department of Biology, Queens, New York, USA

**Keywords:** largemouth bass virus, *Micropterus salmoides*, sequencing

## Abstract

We report the coding-complete genome sequence of a largemouth bass virus isolate obtained from diseased largemouth bass (*Micropterus salmoides*) in China. The genome is 99,378 bp in length with a GC content of 52.1% and contains 102 predicted coding DNA sequences.

## ANNOUNCEMENT

*Ranaviruses* are double-stranded DNA viruses in the family *Iridoviridae* that infect a wide range of fish species (1, 2). Largemouth bass virus (LMBV) is a *Ranavirus* pathogen of largemouth bass (*Micropterus salmoides*) (3). Here, we report the coding-complete genome sequence of the LMBV isolate SYSUMS-2025.

The LMBV isolate whose coding-complete genome sequence is reported here (GenBank accession number: PX879566) was derived from diseased largemouth bass collected in Guangdong Province, China (23.479°N, 113.323°E), in 2025. Since no live animals were experimentally manipulated or sacrificed for this study, ethical approval was not required. Spleen tissues from diseased fish were homogenized in phosphate-buffered saline (PBS; pH 7.4), clarified by centrifugation, and inoculated onto the mandarin fish (*Siniperca knerii*) brain cell line (passage 31) for virus isolation as previously described (2). Subsequently, the virus was propagated in this cell line, harvested after cytopathic effects were observed, and concentrated via ultrafiltration. Total nucleic acids used for PCR and sequencing were extracted using the FastPure Viral DNA/RNA Mini Kit Pro (Vazyme), and the viral identity was confirmed by PCR amplification of the major capsid protein (MCP) gene using LMBV-specific primers (F: 5′-GCGGCCAACCAGTTTAACGCAA-3′; R: 5′-AGGACCCTAGCTCCTGCTTGAT-3′) with 2× Taq PCR Master Mix (Vazyme, China). PCR cycling conditions were 95°C for 3 min; 25 cycles of 95°C for 30 s, 55°C for 30 s, and 72°C for 30 s; and 72°C for 3 min.

Whole-genome sequencing was performed using Oxford Nanopore Technologies (ONT) and Illumina platforms. A sequencing library was prepared using the SQK-NBD114.96 Kit (Oxford Nanopore Technologies). Sequencing was conducted on a PromethION platform using an R10.4.1 Flow Cell. Base calling, adapter trimming, and quality filtering were performed using Guppy (v6.5.7) (4). A total of 27,522 raw reads were generated, with an N50 read length of 9,607 bp. Host-derived reads were removed by mapping ONT reads to the *Siniperca knerii* reference genome (GenBank accession no. GCF_020085105.1) using minimap2 (v2.26-r1175) (5). The final assembly achieved an average genome coverage of 1,893.4×. For Illumina sequencing, DNA libraries were prepared using the VAHTS Universal Plus DNA Library Prep Kit for Illumina V2 (Vazyme), and paired-end sequencing (2 × 150 bp) was performed on an Illumina NovaSeq 6000 platform. After quality control and host read removal, 226.12 Mb of Illumina data were retained. Read trimming and adapter removal were performed using fastp (v0.20.0) with a Q20 threshold (6). *De novo* genome assembly was performed using ONT long reads in Flye (v2.9.2-b1786) (7). The draft assembly was subsequently polished using Illumina reads with Pilon (v1.24) (8). Coding DNA sequences were predicted and annotated using Prokka (v1.14.6) (9). Default parameters were used unless otherwise specified.

**Fig 1:**
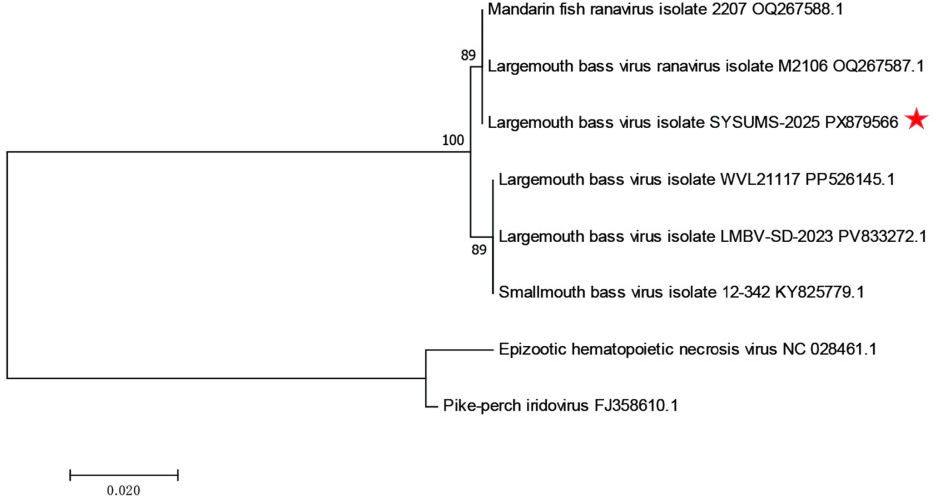
Maximum-likelihood phylogenetic tree based on amino acid sequences of the major capsid protein (MCP) of largemouth bass virus. Reference sequences were retrieved from the National Center for Biotechnology Information (NCBI) GenBank database using BLAST searches (accessed January 2026). Multiple sequence alignment was performed using MUSCLE implemented in MEGA7 (v7.0.26). The phylogenetic tree was inferred using the maximum-likelihood method with 1,000 bootstrap replicates in MEGA7 and visualized in the same software. Epizootic hematopoietic necrosis virus and pike-perch iridovirus were used as outgroups. The isolate SYSUMS-2025 obtained in this study is indicated by a red asterisk.

The coding-complete genome of the LMBV isolate was assembled into a single linear contig of 99,378 bp, with a GC content of 52.1%. A total of 102 coding DNA sequences were predicted. BLASTn analysis using default parameters showed that the genome shared up to 99.89% nucleotide identity with previously reported LMBV genomes, including strain M2106 (GenBank accession no. OQ267587.1) (10). Phylogenetic analysis based on MCP amino acid sequences in MEGA7 showed that the SYSUMS-2025 isolate clustered within the LMBV clade together with other known LMBV isolates (Fig. 1) (11).

## Data Availability

The complete genome sequence of largemouth bass virus has been deposited in GenBank under accession number PX879566. The Oxford Nanopore long-read sequencing data and Illumina short-read sequencing data have been deposited in the Sequence Read Archive under accession numbers SRR36829122 and SRR36864002, respectively, under BioProject PRJNA1402781 and BioSample SAMN54605172.
